# Supplementary material for: A non-mosaic transchromosomic mouse model of Down syndrome carrying the long arm of human chromosome 21
Source: eLife. 2020 Jun 29;9:e56223. doi: 10.7554/eLife.56223 (PMC7358007; doi:10.7554/eLife.56223)
Supplement: Figure 2—source data 2. — HSA21 expression pattern in P1 TcMAC21 brain, source data. [file elife-56223-fig2-data2.docx]

**Figure 2–Source Data 2. Effects of HSA21 on gene expression of other mouse chromosomes analyzed by RNA-seq.**
