# Supplementary figures and images for: A non-mosaic transchromosomic mouse model of Down syndrome carrying the long arm of human chromosome 21
Source: eLife. 2020 Jun 29;9:e56223. doi: 10.7554/eLife.56223 (PMC7358007; doi:10.7554/eLife.56223)

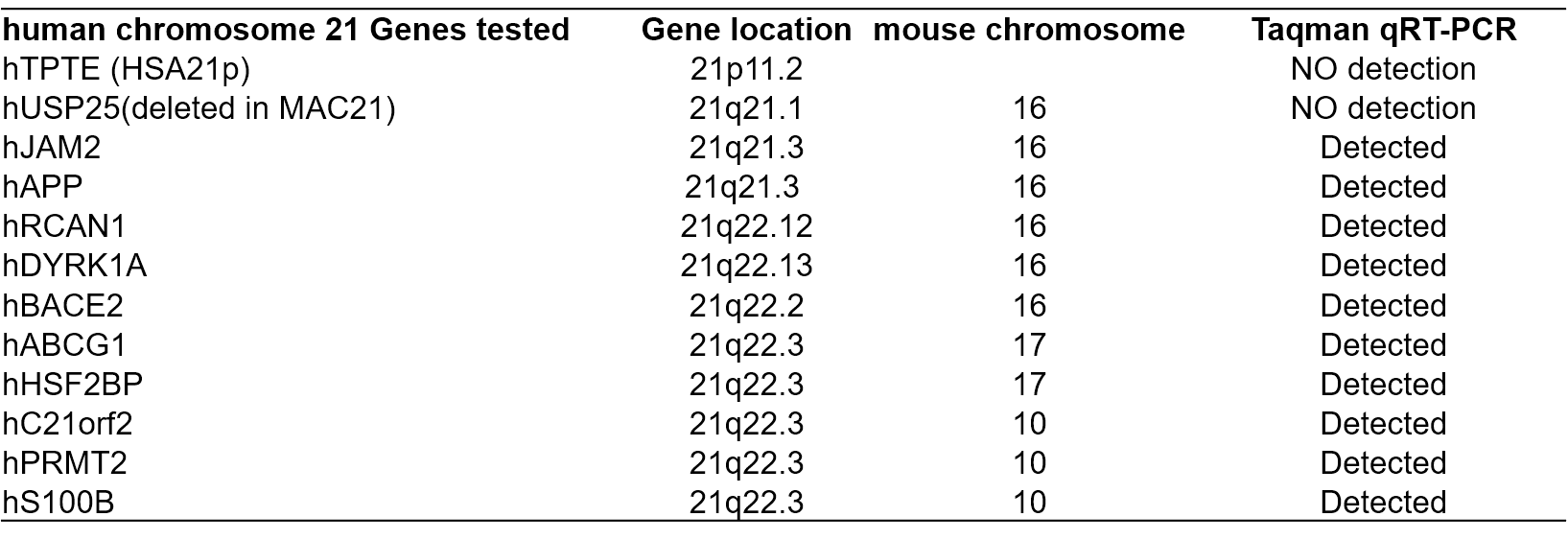


**Figure 3–Source Data 1. HSA21 genes were tested in TcMAC21 using human specific Taqman assay**

Supplement: Figure 3—source data 1. [file elife-56223-fig3-data1.docx]
