# Supplementary material for: A non-mosaic transchromosomic mouse model of Down syndrome carrying the long arm of human chromosome 21
Source: eLife. 2020 Jun 29;9:e56223. doi: 10.7554/eLife.56223 (PMC7358007; doi:10.7554/eLife.56223)
Supplement: Figure 4—source data 1. [file elife-56223-fig4-data1.docx]

**Figure 4–Source Data 1. Absolute volume of brain structures in TcMAC21 and Eu were analyzed by MRI**


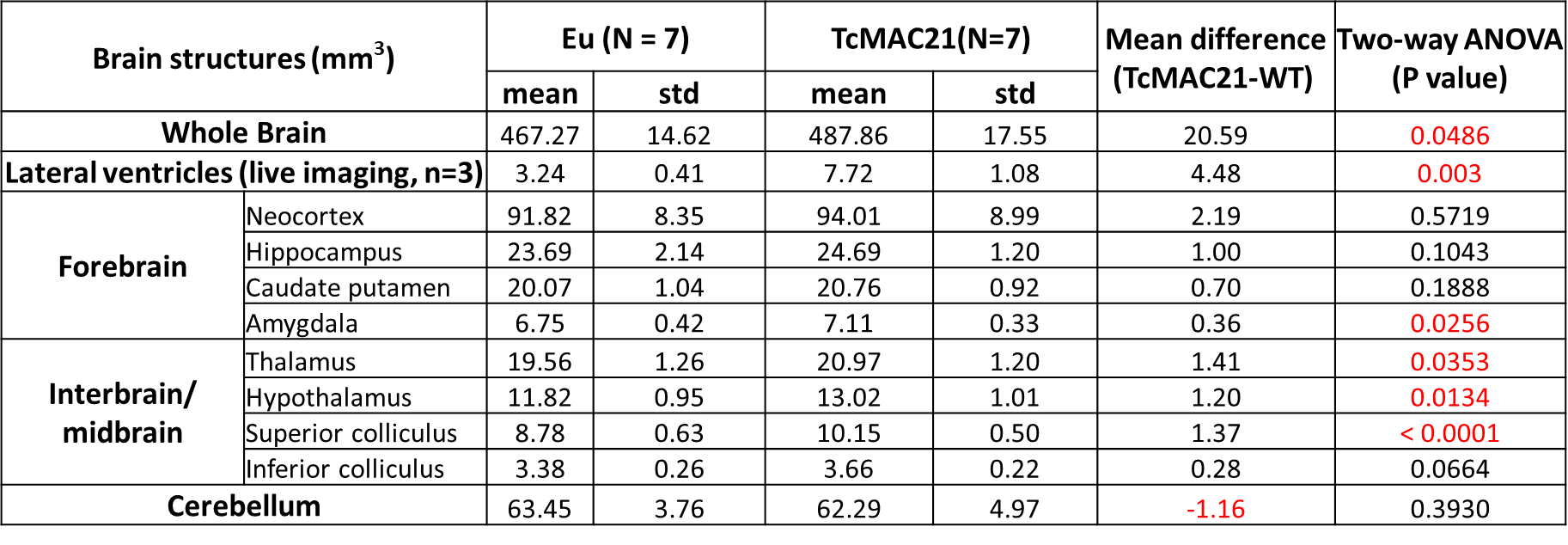


* Segmentation of brain, hippocampus, cerebellum and lateral ventricle were manually verified, and data were analyzed by two-way ANOVA. Except the lateral ventricle using *in vivo* MRI data only, data of all other brain structures came from both *in vivo* and *ex vivo* MRI. Therefore, even though we found that the genetic effect was only minimally affected by MRI methods, it was still more appropriate to use two-way ANOVA than one-way ANOVO or t-tests as we needed to consider both genetic factor (Eu and TcMAC21) and method factor (*in vivo* and *ex vivo* MRI).
