# Supplementary material for: A non-mosaic transchromosomic mouse model of Down syndrome carrying the long arm of human chromosome 21
Source: eLife. 2020 Jun 29;9:e56223. doi: 10.7554/eLife.56223 (PMC7358007; doi:10.7554/eLife.56223)
Supplement: Figure 4—source data 2. [file elife-56223-fig4-data2.docx]

**Figure 4–Source Data 2. Percentage of brain volume in TcMAC21 and Eu were analyzed by MRI**


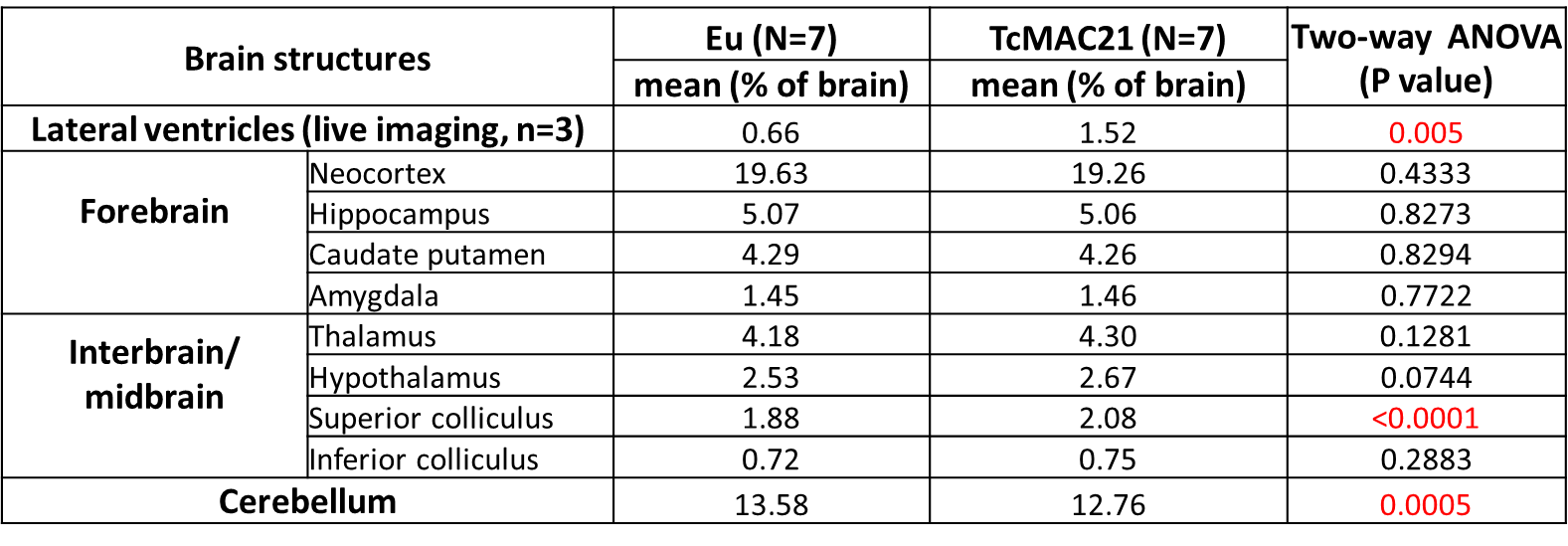


* Segmentation of brain, hippocampus, cerebellum and lateral ventricle were manually verified, and data were analyzed by two-way ANOVA.
