# Supplementary material for: A non-mosaic transchromosomic mouse model of Down syndrome carrying the long arm of human chromosome 21
Source: eLife. 2020 Jun 29;9:e56223. doi: 10.7554/eLife.56223 (PMC7358007; doi:10.7554/eLife.56223)
Supplement: Figure 5—source data 1. [file elife-56223-fig5-data1.docx]

**Figure 5–Source Data 1. Peripheral blood analyses in TcMAC21 and Eu**


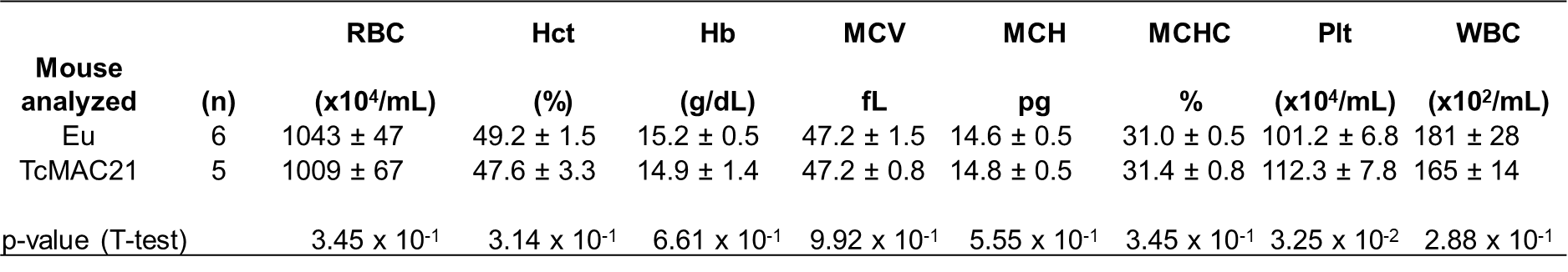


*Data were analyzed by two-tailed t-test and expressed as mean ± SD.
