# Supplementary material for: A non-mosaic transchromosomic mouse model of Down syndrome carrying the long arm of human chromosome 21
Source: eLife. 2020 Jun 29;9:e56223. doi: 10.7554/eLife.56223 (PMC7358007; doi:10.7554/eLife.56223)
Supplement: Figure 6—source data 1. [file elife-56223-fig6-data1.docx]

**Figure 6–Source Data 1. Husbandry in TcMAC21**


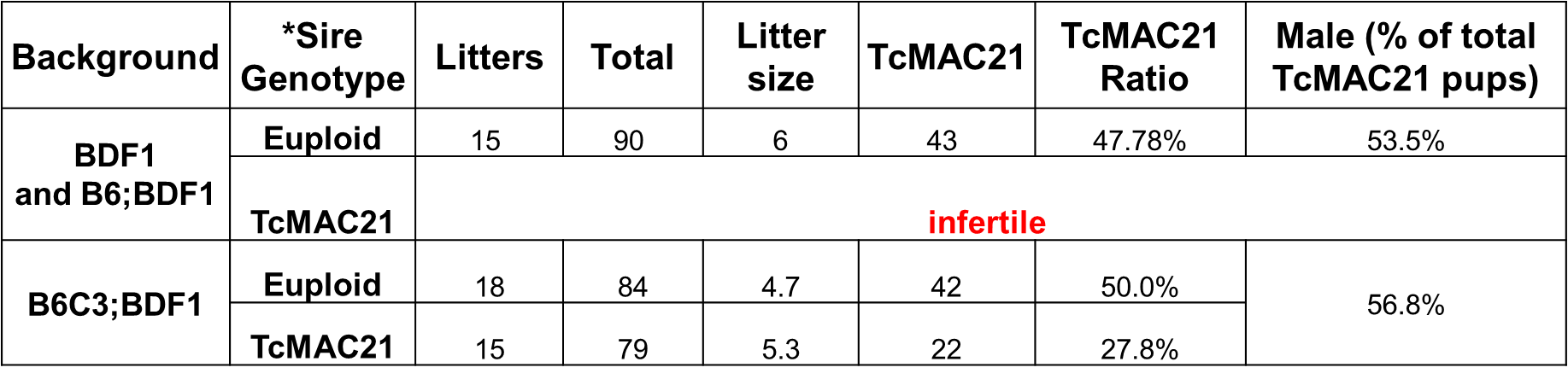


* Sire genotype means the genotype of male in mating cages. For example, if sire genotype is “euploid”, there are an euploid male and TcMAC21 female(s) in the same mating cage.
